# Quantitative ultrasound fatty liver evaluation in a pediatric population: comparison with magnetic resonance imaging of liver proton density fat fraction

**DOI:** 10.1007/s00247-023-05749-9

**Published:** 2023-09-12

**Authors:** Giorgia Polti, Francesco Frigerio, Giovanni Del Gaudio, Patrizia Pacini, Vincenzo Dolcetti, Maurizio Renda, Sergio Angeletti, Michele Di Martino, Giovanni Iannetti, Francesco Massimo Perla, Eleonora Poggiogalle, Vito Cantisani

**Affiliations:** 1 Department of Radiological Sciences, Oncology and Pathology, Sapienza University of Rome, Rome, Italy; 2 Department of Experimental Medicine, Sapienza University of Rome, Piazzale Aldo Moro 5, 00185 Rome, Italy; 3 Department of Pediatrics, Sapienza University of Rome, Rome, Italy

**Keywords:** Fatty liver disease, Magnetic resonance imaging, Non-alcoholic fatty liver disease, Pediatrics, Ultrasound

## Abstract

**Background:**

Biopsy remains the gold standard for the diagnosis of hepatic steatosis, the leading cause of pediatric chronic liver disease; however, its costs call for less invasive methods.

**Objective:**

This study examined the diagnostic accuracy and reliability of quantitative ultrasound (QUS) for the assessment of liver fat content in a pediatric population, using magnetic resonance imaging proton density fat fraction (MRI-PDFF) as the reference standard.

**Materials and methods:**

We enrolled 36 patients. MRI-PDFF involved a 3-dimensional T2*-weighted with Dixon pulse multiple-echo sequence using iterative decomposition of water and fat with echo asymmetry and least squares estimation (IDEAL IQ). QUS imaging relied on the ultrasound system “RS85 A” (Samsung Medison, Seoul, South Korea) and the following software: Hepato-Renal Index with automated region of interest recommendation (EzHRI), Tissue Attenuation Imaging (TAI), and Tissue Scatter Distribution Imaging (TSI). For each QUS index, receiver operating characteristic (ROC) curve analysis against MRI-PDFF was used to identify the associated cut-off value and the area under the ROC curve (AUROC). Concordance between two radiologists was assessed by intraclass correlation coefficients (ICCs) and Bland–Altman analysis.

**Results:**

A total of 61.1% of the sample (*n*=22) displayed a MRI-PDFF ≥ 5.6%; QUS cut-off values were TAI=0.625 (AUROC 0.90, confidence interval [CI] 0.77–1.00), TSI=91.95 (AUROC 0.99, CI 0.98–1.00) and EzHRI=1.215 (AUROC 0.98, CI 0.94–1.00). Inter-rater reliability was good-to-excellent for EzHRI (ICC 0.91, 95% C.I. 0.82–0.95) and TAI (ICC 0.94, 95% C.I. 0.88–0.97) and moderate to good for TSI (ICC 0.73; 95% C.I. 0.53–0.85).

**Conclusion:**

Our results suggest that QUS can be used to reliably assess the presence and degree of pediatric hepatic steatosis.

**Graphical abstract:**

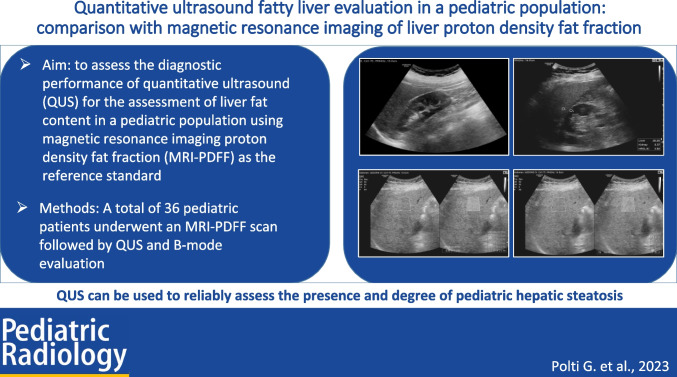

**Supplementary information:**

Supplementary material is available at 10.1007/s00247-023-05749-9.

## Introduction

Nonalcoholic fatty liver disease (NAFLD) is the main form of chronic liver disease in children and adolescents. Obesity and associated metabolic disorders are the staple risk factors for the development of NAFLD, which may be part of the comprehensive clinical picture of metabolic syndrome. With the increasing prevalence of childhood obesity, hepatic steatosis will likely become a major public health issue [1]. The estimated prevalence of hepatic steatosis in Europe ranges from 1.3% to 22.5% for children aged 3–18 years, with a prevalence of 11% among adolescents [2].

As far as we are aware, there are no cohort studies giving insight into the course of NAFLD and nonalcoholic steatohepatitis (NASH) in children or their potential evolution into fibrosis and advanced cirrhosis during childhood and early adulthood [3, 4]. The absence of overt clinical manifestations makes precocious NAFLD diagnosis difficult; as a consequence, the diagnosis of pediatric NAFLD is often incidental, at an average age of 11–13 years [4].

Despite the availability of noninvasive methods to confirm a diagnosis of NAFLD, liver biopsy remains the current gold standard for steatosis and NASH classification. However, biopsy has a number of drawbacks: it is invasive and operator-dependent (and thus subject to sampling variability), requires inter-pathologist agreement and imposes a degree of clinical risk which cannot be overlooked [5]. These limitations make liver biopsy impractical in the routine assessment of liver fat content for patients with uncomplicated NAFLD. Liver enzyme assays systematically underestimate the actual prevalence of the disease and display low prognostic value for the development of NASH [6].

Less invasive methods to exclude hepatic steatosis are required not just as a first step in the diagnostic pathway, but also during the follow-up phase, to monitor the impact of any interventions.

Recently, a few studies have been published investigating new ultrasound (US) technologies for quantitative assessment of liver fat content in children [7, 8] with good results; however, optimal threshold values for these techniques have not yet been defined and approved [9]. Currently, magnetic resonance spectroscopy (MRS) and magnetic resonance imaging proton density fat fraction (MRI-PDFF) represent the only noninvasive diagnostic methods for quantifying hepatic steatosis. Conventional B-mode US is a widely used imaging tool for the noninvasive subjective assessment of hepatic steatosis [10]. However, the US evaluation of fatty liver is based on the operator’s expertise; there is a subjective estimation of the extent of fatty infiltration in the liver, with a sensitivity of 60.9–65.0% for the detection of mild steatosis (fat content>5% but <33%) [11]. Compared to histology, the overall sensitivity and specificity of B-mode US were, respectively, 84.8% and 93.6%, with an area under the receiver operating characteristic curve (AUROC) of 0.93 (95% confidence interval [CI] 0.91–0.95) [12].

Despite these limitations, B-mode US is the preferred first-line diagnostic imaging procedure suggested in patients with nonalcoholic fatty liver disease [13].

Recently, novel liver composition assessment techniques (quantitative ultrasound, QUS) have been investigated for their usefulness in the evaluation of hepatic steatosis based on an analysis of the radiofrequency echoes detected by the transducer and on the automatic calculation of the hepato-renal index. The aims of the present study were to assess the diagnostic accuracy and inter-operator variability of QUS and the diagnostic accuracy of B-mode US in pediatric NAFLD using MRI-PDFF as the reference standard.

## Materials and methods

Patients were enrolled between January 2021 and May 2022 at the Department of Pediatrics, Sapienza University of Rome, Italy. The main inclusion criteria were age <18 years and a body mass index (BMI)-for-age>85th percentile (World Health Organization [WHO] growth charts, 5–19 years [14]). The exclusion criteria were the presence of diabetes mellitus (type 1 or 2), thyroid illnesses and liver diseases other than NAFLD. The parents or legal guardians of all patients signed an informed consent form before enrollment. Calibrated scale and stadiometer were used for body weight and height assessment, respectively; waist circumference was taken with an inextensible tape at a point midway between the inferior margin of the lowest rib and the iliac crest [15]. Obesity prevalence was assessed using both the WHO [16] and International Obesity Task Force (IOTF) [17] definitions.

Resting blood pressure was measured by auscultation using a pediatric aneroid non-mercury manometer, with the patient maintaining a seated position for 5 min before evaluation.

The following biochemical parameters were measured using commercial assays: total cholesterol, HDL-cholesterol, LDL-cholesterol, non-HDL-cholesterol, triglyceride, chylomicron remnants, basal glucose and basal insulin. After collecting fasting samples, a standard oral glucose tolerance test was performed and blood samples withdrawn after 2 h; 2-h glucose was measured. Homeostasis model assessment of insulin resistance was calculated from fasting plasma insulin and glucose levels using the formula: insulin×glucose/405 (mU/L×mg/dL) [18].

All participants underwent MRI, B-mode US and QUS using the following software: EzHRI™ (Hepato-Renal Index with automated region of interest [ROI] recommendation), TAI™ (Tissue Attenuation Imaging), and TSI™ (Tissue Scatter Distribution Imaging) (all software, Samsung Medison, Hongcheon, Republic of Korea).

Participants were asked to fast at least 6 h before the B-mode US. An experienced radiologist (V.C. with over 25 years of experience) and a radiologist (G.P., with four years of training) performed a conventional grayscale ultrasound with QUS imaging using a clinical US system (RS85 A, Samsung Medison, Seoul, South Korea) with a curved array transducer.

Routine B-mode US images of the liver were obtained using subcostal and intercostal planes and stored. Hepatic steatosis was diagnosed based on known US findings, including increased parenchymal echogenicity, hepatorenal echo contrast, impaired visualization of the diaphragm line and intrahepatic portal vein wall, and deep attenuation of the liver parenchyma [19]. The severity of hepatic steatosis was qualitatively scored on a 4-point scale using the Hamaguchi scoring system [20] with scores of 0, 1, 2, and 3 indicating absent, mild, moderate, and severe steatosis, respectively.

Studies have shown that the hepatorenal index or hepatorenal ratio is a sensitive and noninvasive test [21–23]. It is a simple calculation of the B-mode ratio, or the brightness ratio of the liver parenchyma over the renal cortex in each user-selected ROI. EzHRI functions in much the same way as the conventional hepatorenal index but offers greater convenience and an improved workflow by suggesting initial ROI positions. EzHRI segments the input image into the kidney and liver, based on deep learning technology. It then uses the stochastic analysis method, which extracts three brightness classes from each segmented organ, to extract only the cortex while excluding other structures and artifacts. Lastly, it finds the ROIs in the liver and kidney exhibiting the lowest brightness variance, calculates the average brightness ratio for each ROI, and then measures the heptaorenal index.

TAI is a tool that quantitatively measures attenuation of US signals received from the liver. Attenuation of US signals is due to the gradual loss of signal strength due to absorption, reflection, refraction, and scattering. TAI quantifies attenuation based on changes in the center frequency under the optimal transmission and reception conditions. Attenuation is increased in high-frequency components, resulting in changes in the center frequency of the signal; the severity of fatty liver disease is positively associated with the degree of attenuation [24, 25]. To ensure accurate measurement, it is recommended that the user selects a ROI in the right lobe at the VIII segment, at least 3 cm away from the liver capsule and excluding the major veins, with the patients holding their breath. The reliability of the measurement is expressed as an *R*^2^ value. It is recommended that the user performs a measurement in a region with an *R*^2^ value of 0.6 or above.

TSI is a tool that quantifies the scattered signal distribution based on backscattered signals. Scattering, the reflection of ultrasonic waves in multiple directions is not affected by their entry angle and produces speckle patterns in US images. This changes the distribution of backscattered US signals, based on the scattering intensity. The distribution can be modeled by applying a statistical distribution. The Rayleigh distribution is used to represent the case of a high density of random scatterers without coherent signal components. Based on this model, TSI represents scattering by quantifying the correlation between the backscattered signals and the Rayleigh distribution [25–27].

MRI scans were acquired using a 3-tesla magnet (GE Discovery 750; General Electric Healthcare, Milwaukee, WI) with a peak gradient amplitude of 50 mT/m, a time to peak of 200 μs, and a 32-element body torso-array coil system. For liver fat quantification, the following technique was used: 3-dimensional (D) T2*-weighted with Dixon pulse multiple-echo sequence (MRI-PDFF) with the following parameters: TR, 12.9 ms; TE six different echoes from 1.6 to 9.8 ms field of view, 35–40 cm; matrix, 224×60; bandwidth, 125 kHz; FA, 5°; section thickness, 5 mm with 28 sections, acquisition time 25 s. The images of the iterative decomposition of water and fat with echo asymmetry and least squares estimation (IDEAL Q) sequence were processed using the software provided by the manufacturer (IDEAL-IQ; General Electric Healthcare) to create water, fat, in-phase, out-of-phase, R2* and fat fraction maps instantaneously [5]. Quantification of liver steatosis from the IDEAL sequence was performed by drawing a 2-cm^2^ ROI in all liver segments, based on the software-generated fat fraction map [28].

Data were collected in a Microsoft Excel-format spreadsheet. Statistical calculations were performed using SPSS software (IBM SPSS Statistics for Windows, IBM Corp., Version 28.0. Armonk, NY), with the exception of Bland-Altman analysis (including plots) which was performed using jamovi (The jamovi project, Version 2.3, Sydney, Australia) [29]. To assess normality, variables were checked in terms of skewness and kurtosis and the Shapiro-Wilk normality test was performed; variables that were not normally distributed underwent appropriate numeric transformation (logarithmic, squared root, or reciprocal functions). After categorizing patients according to the absence (MRI-PDFF<5.6%) or presence (MRI-PDFF≥5.6%) of hepatic steatosis, baseline comparisons between the two groups were performed using Mann-Whitney *U* test. To calculate the diagnostic performance of TAI, TSI and EzHRI in detecting patients with steatosis defined by MRI-PDFF, ROC curve analysis was performed; optimal cut-off values were defined as those maximizing the Kolmogorov-Smirnov statistic. Agreement between B-mode US and MRI-PDFF in differentiating patients with and without liver steatosis was assessed by means of unweighted Cohen’s kappa (*κ*). The *κ* scores were interpreted using Landis and Koch’s criteria [30]. The levels of concordance between the two radiologists using TAI, TSI, and EzHRI were expressed with a two-way mixed-effects single-measure intraclass correlation coefficient for agreement (ICC); ICCs were reported in accordance with Koo et al. [31]. Bland-Altman plots were drawn and mean bias, upper and lower limits of agreement and their 95% CI were plotted. *P*-values of less than 0.05 were considered statistically significant.

## Results

Out of the 36 children and adolescents recruited in the study (17 females), MRI showed a PDFF value ≥5.6% in 22 patients (males: *n*=13 [59%]). Obesity prevalence ranged from 66.6% (*n*=24) to 44.4% (*n*=16), whereas overweight affected 33.3% (*n*=12) to 55.5% (*n*=20) of the sample (IOTF and WHO definitions, respectively). The general characteristics of the study sample, categorized according to hepatic steatosis status, are expressed as medians (1st–3rd quartile) in Table 1.

**Table 1:** Stratification of biochemical and anthropometric characteristics in patients with and without evidence of hepatic steatosis based on magnetic resonance imaging proton density fat fraction (MRI-PDFF). No statistically significant between-group difference was apparent (Mann–Whitney *U* test; all *P*> 0.1)

Variable (units)	MRI-PDFF < 5.6%	MRI-PDFF ≥ 5.6%	*P*-value
	***n***=14	***n***=22	
Age (years)	13 (11–14)	11 (10–13)	0.12
BMI (kg/m^2^)	27.9 (26.2–28.8)	26.2 (23.3–31.4)	0.71
WC (cm)	88.0 (72.4–91.5)	92.3 (78.1–98.9)	0.45
WC (percentile)	95 (50–97)	97.0 (90–97)	0.56
TOT-C (mg/dL)	166 (146.3–188.3)	179.0 (138.8–198.5)	0.49
LDL-C (mg/dL)	89.5 (78.3–114.5)	105.0 (81.5–128.5)	0.41
HDL-C (mg/dL)	48.0 (40.8–54.0)	49.5 (42.3–57.3)	0.76
non-HDL-C (mg/dL)	113.5 (85.5–134.3)	121.0 (99.8–152.8)	0.28
CMr (mg/dL)	19.5 (10.8–30.7)	19.0 (13.5–25.7)	0.91
TG (mg/dL)	79.5 (55.0–114.0)	95.5 (63.7–130.5)	0.48
Glu_0′_ (mg/dL)	85.5 (83.0–90.5)	85.5 (81.0–90.0)	0.59
Ins_0′_ (mUI/mL)	21.0 (13.1–24.3)	18.7 (13.6–44.0)	0.81
Glu_120′_ (mg/dL)	101.0 (87.5–110.5)	106.0 (91.0–115.5)	0.33
HbA1c (%)	5.3 (5.0–5.4)	5.3 (5.0–5.5)	0.71
HOMA-IR	4.58 (2.73–5.01)	3.80 (2.85–7.01)	0.89
SBP (mmHg)	119 (111–124)	117 (110–121)	0.60
DBP (mmHg)	70 (65–71)	70 (67–76)	0.19

*BMI* body mass index, *CMr* chylomicron remnants, *DBP* diastolic blood pressure, *Glu*_*0*′_ fasting glucose, *Glu*_*120*′_ 2-h glucose after oral glucose tolerance test (OGTT), *HbA1c* glycosylated hemoglobin, *HDL-C* high-density lipoprotein cholesterol, *Ins*_*0*′_ fasting insulin, *LDL-C* low-density lipoprotein cholesterol, *non-HDL-C* non-HDL cholesterol, *SBP* systolic blood pressure, *TG* triglycerides, *TOT-C* total cholesterol, *WC* waist circumference

**Fig. 1:**
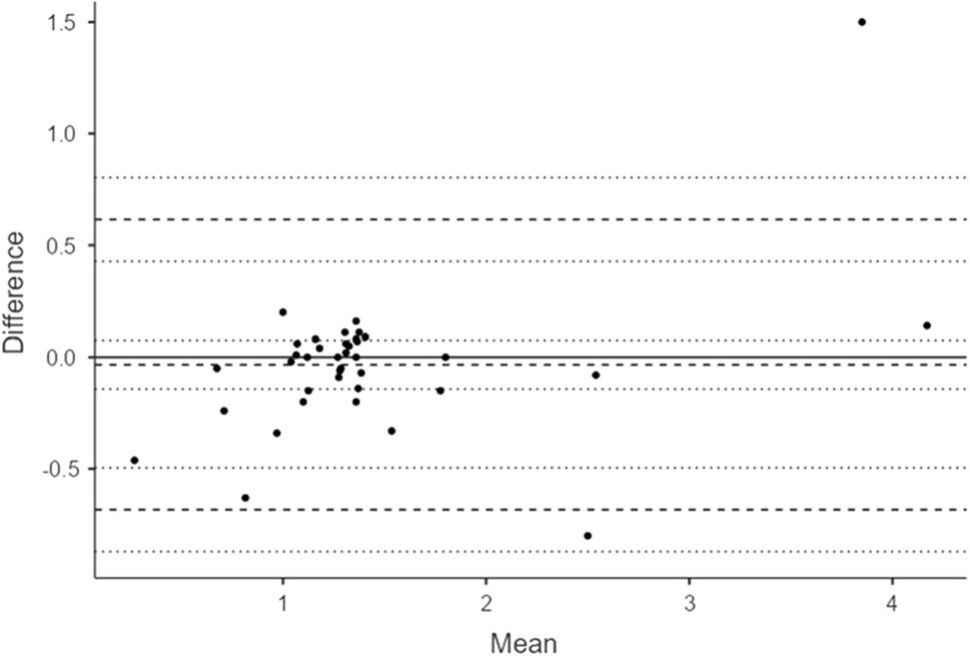
Bland–Altman plot of hepato-renal index with automated region of interest recommendation values (1st vs. 2nd radiologist). *Solid horizontal line* *y*=0, *broken lines* mean difference (*Δ*), upper and lower limits of agreement (LoA), *dotted lines* 95% confidence interval for mean *Δ*, upper LoA and lower LoA. See Supplementary Material 1 for further details

**Fig. 2:**
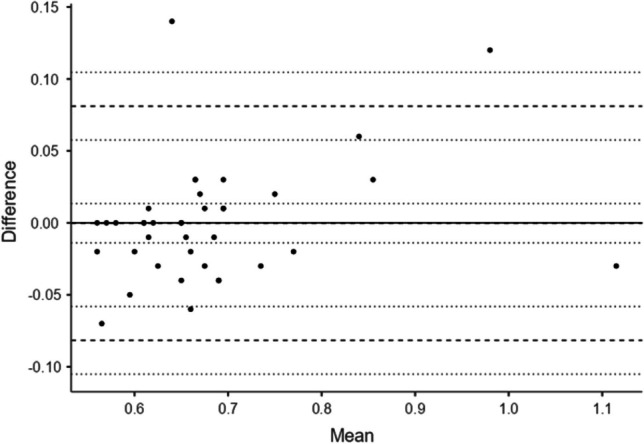
Bland–Altman plot of Tissue Attenuation Imaging values (1st vs. 2nd radiologist). *Solid horizontal line* *y*=0, *broken lines* mean difference (*Δ*), upper and lower limits of agreement (LoA), *dotted lines* 95% confidence interval for mean *Δ*, upper LoA and lower LoA. See Supplementary Material 2 for further details

**Fig. 3:**
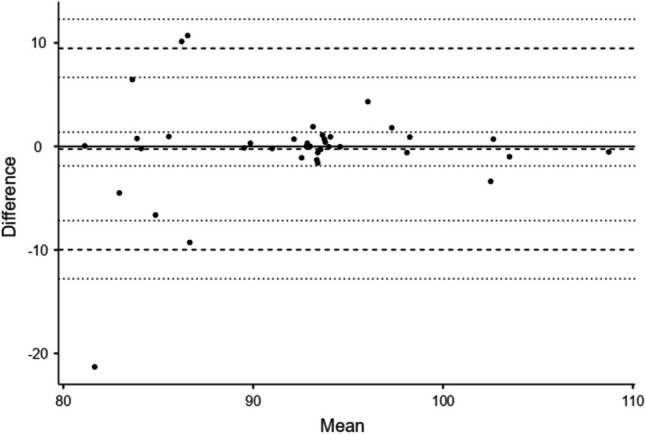
Bland–Altman plot of Tissue Scatter Distribution Imaging values (1st vs. 2nd radiologist). *Solid horizontal line* *y*=0, *broken lines* mean difference (*Δ*) upper and lower limits of agreement (LoA), *dotted lines* 95% confidence interval for mean *Δ*, upper LoA and lower LoA. See Supplementary Material 3 for further details

The level of agreement between B-mode US and MRI-PDFF definition of hepatic steatosis was very good for the 1st radiologist (*κ*=0.94, 95% CI 0.83–1.00) and good-to-very good for the 2nd radiologist (*κ*=0.82, 95% CI 0.62–1.00). Inter-operator variability of the QUS software was good-to-excellent for EzHRI (ICC 0.91, 95% CI 0.82–0.95) and TAI (ICC 0.94, 95% CI 0.88–0.97) and moderate-to-good for TSI (ICC 0.73; *p*<0.001; 95% CI 0.53–0.85). Bland-Altman plots for each QUS index are displayed in Figs. 1, 2, and 3; mean difference (mean *Δ*), limits of agreement and 95% CI are reported in Supplementary Material S1–S3.

**Fig. 4:**
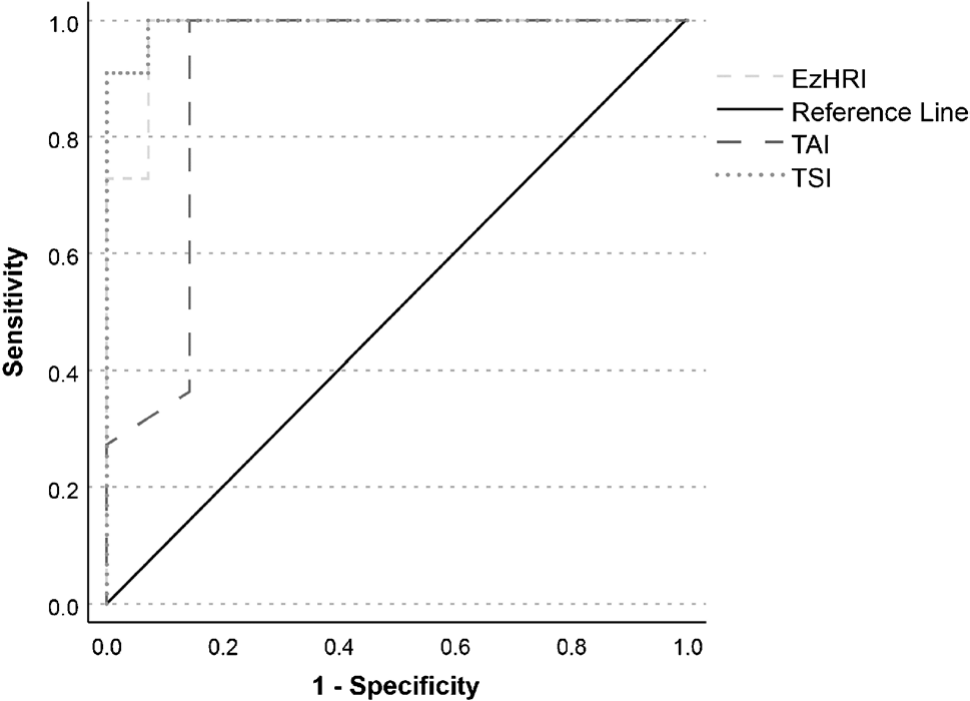
Receiver operating characteristic curve analysis of TSI, TAI and EzHRI against MRI-PDFF ≥ 5.6% (reference method). *EzHRI* Hepato-Renal Index with automated region of interest (ROI) recommendation, *MRI-PDFF *magnetic resonance imaging proton density fat fraction, *TAI *Tissue Attenuation Imaging, *TSI* Tissue Scatter Distribution Imaging

Using ROC curve analysis, the diagnostic accuracy of QUS methods (values obtained by the 1st radiologist) was investigated against MRI-PDFF (reference method) (Fig. 4). The computed AUROC were 0.98 (95% CI 0.94–1.00) for EzHRI, 0.90 (95% CI 0.77–1.00) for TAI and 0.99 (95% CI 0.98–1.00) for TSI. Data on diagnostic performance of TAI, EzHRI and TSI are given in Table 2.

**Table 2:** Diagnostic performance of TSI, TAI, and EzHRI using receiving operating characteristic curve analysis

	TSI	TAI	EzHRI
Cut-off value	≥ 91.95	≥ 0.625	≥ 1.215
AUROC	0.994 (0.976–1.000)	0.903 (0.773–1.000)	0.981 (0.939–1.000)
Sensitivity	100%	100%	100%
Specificity	92.9%	85.7%	92.9%
Likelihood ratio +	14.1	7.0	14.1
Likelihood ratio -	< 0.01	<0.01	<0.01

*AUROC *area under the receiver operating characteristic curve,* EzHRI* Hepato-Renal Index with automated region of interest (ROI) recommendation, *TAI* Tissue Attenuation Imaging, *TSI* Tissue Scatter Distribution Imaging

## Discussion

In our study, the sensitivity and specificity of EzHRI were compared with MRI-PDFF, resulting in an AUROC of 0.98 (0.94–1.00). With regard to TSI, several studies have shown significantly better diagnostic performance in the detection of liver fat content >10%, compared to qualitative visual evaluation in B-mode [32]. Thus, TSI can be considered a valid marker for the identification of the presence of hepatic steatosis in children and adolescents, with an AUROC of 0.99 (0.98–1.00) and a superior diagnostic accuracy to TAI and EzHRI. To date, few studies have focused on the aforementioned QUS indices in the assessment of pediatric liver steatosis; one prospective study evaluated the performance of a different attenuation coefficient (i.e., attenuation imaging, ATI) compared to MRI-PDFF [7], while a cross-sectional study evaluated the diagnostic concordance between ATI and B-mode US [8]. Another recent prospective study included four US technologies for the quantitative assessment of liver fat content in children (the acoustic attenuation coefficient, the hepatorenal index, the Nakagami parameter and the shear wave elastography parameter) using MRI-PDFF as the reference standard. The attenuation coefficient and hepatorenal index accurately detected and quantified liver fat in this small sample of children [33].

Authors of one study assessed the diagnostic performance of quantitative liver echogenicity measurement (hepatorenal index) for hepatic steatosis in a pediatric cohort using MRI-PDFF as the reference method and showed moderate diagnostic performance and a positive correlation with MRI-PDFF with an optimal hepatorenal index cutoff value of >1.75. Furthermore, the incorporation of BMI-percentile and standard deviation of the grayscale echogenicity improved diagnostic performance [34].

In previous studies involving an adult population, an EzHRI value of 1.2 was used as the optimal cut-off (sensitivity: 100%) to detect steatosis [21]. In agreement with these results, this current study in pediatric patients found a similar value for EzHRI (AUROC 0.98, specificity 92.9%) as the best cut-off value. A limitation of our study is the small sample size, which limited our ability to discern different stages of hepatic steatosis; nonetheless, the homogeneity of sex representation, our focus on a pediatric population and the availability of a reference method (MRI-PDFF) counterbalance this drawback. As the operator-dependency of B-mode US assessment represents a major issue, the present study explored the inter-rater reliability of QUS techniques, which was good-to-excellent for EzHRI and TAI and moderate-to-good for TSI.

In a prospective study, US was 90% sensitive in detecting steatosis in 20% of hepatocytes but became less sensitive for lower degrees of fat content [35]. Hepatic steatosis is often homogeneously distributed in the liver, but 10–15% exhibit an atypical heterogeneous distribution. Areas of focal steatosis appear hyperechogenic, most often located near the gallbladder or adjacent to the falciform ligament, sometimes diffuse with typical map-like distribution. In contrast, hypoechogenic areas are termed focal sparing areas and represent different concentrations of steatosis.

From a practical standpoint, an US image indicating steatosis implies the presence of at least 20% of hepatic fat. However, below that threshold, steatosis cannot be reliably detected by standard B-mode US. 

However, classification of steatosis with US is arduous because the operator must subjectively (qualitatively) assess the amount of fatty infiltration. Especially in mild forms of steatosis, B-mode US displays limited accuracy in assessing mild changes of steatosis over time [10].

Therefore, although B-mode US can offer a fairly accurate diagnosis in cases of moderate-to-severe steatosis (defined histologically as ≥ 30% or 33%), with a sensitivity ranging from 81.8 to 100.0% and a specificity of approximately 98% [36], it has several limitations in identifying mild steatosis (fat content >5% but <33%), with a sensitivity ranging from 60.9 to 65.0% [11]. In short, the qualitative assessment of B-mode US is strongly influenced by the severity of hepatic steatosis, the presence of any coexisting liver disease and the experience of the operator, and from these observations, it follows that it is important to develop US methods to accurately quantify hepatic steatosis.

## Conclusion

In conclusion, our results show acceptable reliability for the included QUS methods; furthermore, using MRI-PDFF as the reference method, we propose EzHRI, TAI and TSI cut-off values to diagnose NAFLD in a pediatric setting. These preliminary results require confirmation by further studies investigating the performance of QUS in hepatic steatosis grading. However, the wide availability of QUS software, its ease of use and speed of execution make it a potentially useful tool for the screening and clinical follow-up of pediatric hepatic steatosis.

### Supplementary information

Below is the link to the electronic supplementary material.

Supplementary file1 (DOCX 18 KB)

## Data Availability

The datasets generated during and/or analyzed during the current study are not publicly available due to data protection considerations, but are available from the corresponding author on reasonable request.

## References

[1] Eslam M, Newsome PN, Sarin SK (2020). A new definition for metabolic dysfunction-associated fatty liver disease: an international expert consensus statement. J Hepatol.

[2] Wiegand S, Keller KM, Röbl M (2010). Obese boys at increased risk for nonalcoholic liver disease: evaluation of 16 390 overweight or obese children and adolescents. Int J Obesity.

[3] Panera N, Barbaro B, Della Corte C (2018). A review of the pathogenic and therapeutic role of nutrition in pediatric nonalcoholic fatty liver disease. Nutr Res.

[4] Mann JP, De Vito R, Mosca A (2016). Portal inflammation is independently associated with fibrosis and metabolic syndrome in pediatric nonalcoholic fatty liver disease. Hepatology.

[5] Idilman IS, Aniktar H, Idilman R, et al (2013) Hepatic steatosis: quantification by proton density fat fraction with MR imaging versus liver biopsy. 267:767–775 10.1148/radiol.13121360 23382293

[6] Eslam M, Sanyal AJ, George J (2020). MAFLD: a consensus-driven proposed nomenclature for metabolic associated fatty liver disease. Gastroenterology.

[7] Bulakci M, Ercan CC, Karapinar E (2023). Quantitative evaluation of hepatic steatosis using attenuation imaging in a pediatric population: a prospective study. Pediatr Radiol.

[8] Dardanelli EP, Orozco ME, Oliva V et al (2023) Ultrasound attenuation imaging: a reproducible alternative for the noninvasive quantitative assessment of hepatic steatosis in children. Pediatr Radiol 1–11 10.1007/s00247-023-05601-0 10.1007/s00247-023-05601-0 36869263

[9] Koot BGP, Van Der Baan-Slootweg OH, Bohte AE (2013). Accuracy of prediction scores and novel biomarkers for predicting nonalcoholic fatty liver disease in obese children. Obesity.

[10] Barr RG (2019). Ultrasound of diffuse liver disease including elastography. Radiol Clin North Am.

[11] Ferraioli G, Monteiro LBS (2019). Ultrasound-based techniques for the diagnosis of liver steatosis. World J Gastroenterol.

[12] Hernaez R, Lazo M, Bonekamp S (2011). Diagnostic accuracy and reliability of ultrasonography for the detection of fatty liver: a meta-analysis. Hepatology.

[13] Marchesini G, Day CP, Dufour JF (2016). EASL-EASD-EASO Clinical Practice Guidelines for the management of non-alcoholic fatty liver disease. J Hepatol.

[14] World Health Organization Growth Charts. http://www.who.int/tools/growth-reference-data-for5to19-years/indicators/bmi-for-age. Accessed 6 Sept 2023

[15] Lohman TG, 1940-, Roche AF et al (1988) Anthropometric standardization reference manual. Human Kinetics Books

[16] de Onis M, Onyango AW, Borghi E, Siyam A, Nishida C, Siekmann J. (2007) Development of a WHO growth reference for school-aged children and adolescents. Bull World Health Organ 85:660–667. 10.2471/blt.07.043497 10.2471/BLT.07.043497 PMC2636412 18026621

[17] Cole TJ, Bellizzi MC, Flegal KM, Dietz WH (2000) Establishing a standard definition for child overweight and obesity worldwide: international survey. BMJ 320:1240–1243. 10.1136/bmj.320.7244.1240 10.1136/bmj.320.7244.1240 PMC27365 10797032

[18] Wallace TM, Levy JC, Matthews DR (2004). Use and abuse of HOMA modeling. Diabetes Care.

[19] Ballestri S, Lonardo A, Romagnoli D (2012). Ultrasonographic fatty liver indicator, a novel score which rules out NASH and is correlated with metabolic parameters in NAFLD. Liver Int.

[20] Hamaguchi M, Kojima T, Itoh Y (2007). The severity of ultrasonographic findings in nonalcoholic fatty liver disease reflects the metabolic syndrome and visceral fat accumulation. Am J Gastroenterol.

[21] de Almeida e Borges VF, Diniz ALD, Cotrim HP, et al (2013) Sonographic hepatorenal ratio: a noninvasive method to diagnose nonalcoholic steatosis. J Clin Ultrasound 41:18–25 10.1002/jcu.21994 22997020

[22] Marshall RH, Eissa M, Bluth EI, et al (2012) Hepatorenal index as an accurate, simple, and effective tool in screening for steatosis. 199:997–1002 10.2214/AJR.11.6677 23096171

[23] Lee SS, Park SH (2014). Radiologic evaluation of nonalcoholic fatty liver disease. World J Gastroenterol.

[24] Hyungsuk K, Varghese T (2007). Attenuation estimation using spectral cross-correlation. IEEE Trans Ultrason Ferroelectr Freq Control.

[25] Liao YY, Yang KC, Lee MJ (2016). (2016) Multifeature analysis of an ultrasound quantitative diagnostic index for classifying nonalcoholic fatty liver disease. Scientific Reports.

[26] Mohana Shankar P (2000). A general statistical model for ultrasonic backscattering from tissues. IEEE Trans Ultrason Ferroelectr Freq Control.

[27] Tsui P-H, Wan Y-L (2016) Application of ultrasound Nakagami imaging for the diagnosis of fatty liver. 10.1016/j.jmu.2016.03.005

[28] Di MM, Pacifico L, Bezzi M (2016). Comparison of magnetic resonance spectroscopy, proton density fat fraction and histological analysis in the quantification of liver steatosis in children and adolescents. World J Gastroenterol.

[29] The jamovi project (2023) jamovi (Version 2.3) [Computer Software]. Retrieved from https://www.jamovi.org

[30] Landis JR, Koch GG (1977). The measurement of observer agreement for categorical data. Biometrics.

[31] Koo TK, Li MY (2016). A guideline of selecting and reporting intraclass correlation coefficients for reliability research. J Chiropr Med.

[32] Jeon SK, Lee JM, Joo I, Park SJ (2021). Quantitative ultrasound radiofrequency data analysis for the assessment of hepatic steatosis in nonalcoholic fatty liver disease using magnetic resonance imaging proton density fat fraction as the reference standard. Korean J Radiol.

[33] D’Hondt A, Rubesova E, Xie H (2021). Liver fat quantification by ultrasound in children: a prospective study. Am J Roentgenol.

[34] Frankland MP, Dillman JR, Anton CG (2022). Diagnostic performance of ultrasound hepatorenal index for the diagnosis of hepatic steatosis in children. Pediatr Radiol.

[35] Dasarathy S, Dasarathy J, Khiyami A (2009). Validity of real time ultrasound in the diagnosis of hepatic steatosis: a prospective study. J Hepatol.

[36] Artz NS, Hines CDG, Brunner ST (2012). Quantification of hepatic steatosis with dual-energy computed tomography: comparison with tissue reference standards and quantitative magnetic resonance imaging in the ob/ob mouse. Invest Radiol.

