# Supplementary material for: Quantitative ultrasound fatty liver evaluation in a pediatric population: comparison with magnetic resonance imaging of liver proton density fat fraction
Source: Pediatr Radiol. 2023 Sep 12;53(12):2458–65. doi: 10.1007/s00247-023-05749-9 (PMC10635941; doi:10.1007/s00247-023-05749-9)
Supplement: Supplementary file 1 — Supplementary file1 (DOCX 18 KB) [file 247_2023_5749_MOESM1_ESM.docx]

**Title**

Quantitative ultrasound fatty liver evaluation in a pediatric population: comparison with magnetic resonance imaging of liver proton density fat fraction

**Authors**Giorgia Polti^1^ *, Francesco Frigerio^2^ *, Giovanni Del Gaudio^1^, Patrizia Pacini^1^, Vincenzo Dolcetti^1^, Maurizio Renda^1^, Sergio Angeletti^1^, Michele Di Martino^1^, Giovanni Iannetti^1^, Francesco Massimo Perla^3^, Eleonora Poggiogalle^2^, Vito Cantisani^1^
*equal contribution

**Affiliations**

^1^ Department of Radiological Sciences, Oncology and Pathology, Sapienza University of Rome, Rome, Italy.
^2^ Experimental Medicine Department, Sapienza University of Rome, Rome, Italy
^3^ Department of Pediatrics, Sapienza University of Rome, Rome, Italy.

**Corresponding author**Eleonora Poggiogalle - Experimental Medicine Department, Sapienza University of Rome, Piazzale Aldo Moro 5, 00185, Rome, Italy.
E-mail: [eleonora.poggiogalle@uniroma1.it](mailto:eleonora.poggiogalle@uniroma1.it)

**Supplementary material**

|  | | | | | | | |
| --- | --- | --- | --- | --- | --- | --- | --- |
|  | | | | **95% Confidence Interval** | | | |
|  | | **Estimate** | | **Lower** | | **Upper** | |
| Mean Δ (bias) |  | -0.03 |  | -0.14 |  | 0.08 |  |
| Lower LoA |  | -0.68 |  | -0.87 |  | -0.50 |  |
| Upper LoA |  | 0.62 |  | 0.43 |  | 0.80 |  |
|  | | | | | | | |

**Table S1** Bland-Altman analysis for EzHRI values (1^st^ vs. 2^nd^ radiologist). *EzHRI*, Hepato-renal index with automated ROI recommendation.

|  | | | | | | | |
| --- | --- | --- | --- | --- | --- | --- | --- |
|  | | | | **95% Confidence Interval** | | | |
|  | | **Estimate** | | **Lower** | | **Upper** | |
| Mean Δ (bias) |  | 0.00 |  | -0.01 |  | 0.01 |  |
| Lower LoA |  | -0.08 |  | -0.11 |  | -0.06 |  |
| Upper LoA |  | 0.08 |  | 0.06 |  | 0.10 |  |
|  | | | | | | | |

**Table S2** Bland-Altman analysis for TAI values (1^st^ vs. 2^nd^ radiologist). *TAI*, Tissue Attenuation Imaging.

|  | | | | | | | |
| --- | --- | --- | --- | --- | --- | --- | --- |
|  | | | | **95% Confidence Interval** | | | |
|  | | **Estimate** | | **Lower** | | **Upper** | |
| Mean Δ (bias) |  | -0.25 |  | -1.89 |  | 1.38 |  |
| Lower LoA |  | -9.98 |  | -12.79 |  | -7.17 |  |
| Upper LoA |  | 9.47 |  | 6.66 |  | 12.28 |  |
|  | | | | | | | |

**Table S3** Bland-Altman analysis for TSI values (1^st^ vs. 2^nd^ radiologist). *TSI*, Tissue Scatter Distribution Imaging.
